# Supplementary material for: Hot spot prediction in protein-protein interactions by an ensemble system
Source: BMC Syst Biol. 2018 Dec 31;12(Suppl 9):132. doi: 10.1186/s12918-018-0665-8 (PMC6311905; doi:10.1186/s12918-018-0665-8)
Supplement: Supplementary file 2 — All relevant MATLAB code. A simple Matlab implement of our predictor. (ZIP 19,742 kb) [file 12918_2018_665_MOESM2_ESM.zip › Additional file 2/Read ASA/ASA Property/SKEMPI/1S1QA.txt]

 NetSurfP - Protein Surface Accessibility and Secondary Structure Predictions

  

|  |  |  |
| --- | --- | --- |
|  |  | NetSurfP - Protein Surface Accessibility and Secondary Structure Predictions  Technical University of Denmark |

  


---

```
# For publication of results, please cite:
# A generic method for assignment of reliability scores applied to solvent accessibility predictions.
# Bent Petersen, Thomas Nordahl Petersen, Pernille Andersen, Morten Nielsen and Claus Lundegaard
# BMC Structural Biology 2009, 9:51 doi:10.1186/1472-6807-9-51
#
# Column 1: Class assignment - B for buried or E for Exposed - Threshold: 25% exposure, but not based on RSA
# Column 2: Amino acid
# Column 3: Sequence name
# Column 4: Amino acid number
# Column 5: Relative Surface Accessibility - RSA
# Column 6: Absolute Surface Accessibility
# Column 7: Z-fit score for RSA prediction
# Column 8: Probability for Alpha-Helix
# Column 9: Probability for Beta-strand
# Column 10: Probability for Coil
E V  1S1QA                  1    0.613  94.157  -1.297   0.003   0.003   0.994
E S  1S1QA                  2    0.588  68.878  -0.228   0.058   0.017   0.925
E E  1S1QA                  3    0.483  84.310   0.017   0.782   0.003   0.216
E S  1S1QA                  4    0.689  80.716   0.584   0.858   0.002   0.139
E Q  1S1QA                  5    0.315  56.330   1.206   0.923   0.002   0.076
B L  1S1QA                  6    0.066  12.048   0.044   0.923   0.002   0.076
E K  1S1QA                  7    0.324  66.544   0.919   0.923   0.002   0.076
E K  1S1QA                  8    0.522 107.314   1.347   0.923   0.002   0.076
B M  1S1QA                  9    0.115  23.112   0.217   0.923   0.002   0.076
B V  1S1QA                 10    0.070  10.805   0.010   0.782   0.003   0.216
E S  1S1QA                 11    0.450  52.740   0.291   0.502   0.002   0.495
E K  1S1QA                 12    0.565 116.200   1.546   0.321   0.003   0.675
B Y  1S1QA                 13    0.160  34.192   0.395   0.181   0.016   0.803
E K  1S1QA                 14    0.533 109.535   0.085   0.115   0.016   0.868
E Y  1S1QA                 15    0.291  62.294   0.201   0.109   0.005   0.886
B R  1S1QA                 16    0.251  57.456   0.295   0.502   0.002   0.495
E D  1S1QA                 17    0.632  91.129   0.554   0.694   0.003   0.303
E L  1S1QA                 18    0.232  42.479   1.896   0.879   0.010   0.111
B T  1S1QA                 19    0.015   2.150   1.629   0.938   0.007   0.055
B V  1S1QA                 20    0.105  16.062   1.475   0.975   0.003   0.022
E R  1S1QA                 21    0.474 108.477   1.892   0.970   0.001   0.030
B E  1S1QA                 22    0.117  20.440   1.294   0.970   0.001   0.030
B T  1S1QA                 23    0.016   2.289   1.562   0.988   0.000   0.012
E V  1S1QA                 24    0.264  40.500   1.666   0.988   0.000   0.012
E N  1S1QA                 25    0.418  61.166   1.816   0.988   0.000   0.012
B V  1S1QA                 26    0.030   4.611   0.498   0.988   0.000   0.012
B I  1S1QA                 27    0.074  13.635   0.194   0.970   0.001   0.030
E T  1S1QA                 28    0.545  75.522   0.978   0.923   0.002   0.076
E L  1S1QA                 29    0.486  89.005   1.540   0.782   0.003   0.216
B Y  1S1QA                 30    0.084  17.929   0.768   0.246   0.004   0.750
E K  1S1QA                 31    0.567 116.714   0.588   0.053   0.005   0.942
E D  1S1QA                 32    0.662  95.337   0.909   0.018   0.019   0.964
B L  1S1QA                 33    0.052   9.594   0.170   0.018   0.088   0.893
E K  1S1QA                 34    0.452  92.894   1.199   0.004   0.197   0.799
B P  1S1QA                 35    0.203  28.792  -0.338   0.005   0.262   0.733
E V  1S1QA                 36    0.411  63.140  -0.146   0.005   0.336   0.660
B L  1S1QA                 37    0.287  52.586  -1.173   0.004   0.420   0.576
B D  1S1QA                 38    0.281  40.463   0.191   0.003   0.718   0.279
E S  1S1QA                 39    0.385  45.099   0.295   0.018   0.846   0.136
B Y  1S1QA                 40    0.079  16.775   1.042   0.018   0.846   0.136
B V  1S1QA                 41    0.275  42.298  -0.136   0.003   0.718   0.279
B F  1S1QA                 42    0.122  24.445   0.007   0.004   0.420   0.576
E N  1S1QA                 43    0.632  92.525  -0.792   0.004   0.138   0.858
E D  1S1QA                 44    0.574  82.641  -1.101   0.018   0.047   0.935
E G  1S1QA                 45    0.374  29.473  -1.586   0.053   0.043   0.903
E S  1S1QA                 46    0.411  48.169   0.119   0.056   0.142   0.802
E S  1S1QA                 47    0.303  35.465   0.015   0.074   0.484   0.442
E R  1S1QA                 48    0.285  65.265   0.582   0.087   0.683   0.230
B E  1S1QA                 49    0.147  25.611   0.822   0.023   0.655   0.322
B L  1S1QA                 50    0.062  11.389   0.931   0.018   0.846   0.136
B M  1S1QA                 51    0.022   4.342   0.852   0.011   0.918   0.071
B N  1S1QA                 52    0.096  14.040   0.916   0.006   0.962   0.032
B L  1S1QA                 53    0.022   4.065   0.821   0.001   0.959   0.040
E T  1S1QA                 54    0.273  37.823   0.560   0.001   0.900   0.099
B G  1S1QA                 55    0.057   4.462  -1.408   0.002   0.816   0.182
B T  1S1QA                 56    0.244  33.801  -0.324   0.002   0.816   0.182
B I  1S1QA                 57    0.057  10.527   0.323   0.002   0.816   0.182
B P  1S1QA                 58    0.175  24.804  -0.344   0.002   0.816   0.182
B V  1S1QA                 59    0.055   8.484   0.643   0.001   0.900   0.099
E P  1S1QA                 60    0.285  40.498   0.504   0.002   0.816   0.182
B Y  1S1QA                 61    0.141  30.174  -0.054   0.003   0.718   0.279
E R  1S1QA                 62    0.589 134.927  -0.412   0.004   0.197   0.799
E G  1S1QA                 63    0.698  54.917  -2.260   0.005   0.045   0.951
E N  1S1QA                 64    0.381  55.778  -0.618   0.005   0.336   0.660
E T  1S1QA                 65    0.420  58.226  -0.021   0.004   0.616   0.381
B Y  1S1QA                 66    0.095  20.323   0.184   0.004   0.616   0.381
B N  1S1QA                 67    0.246  36.058  -0.248   0.004   0.514   0.481
B I  1S1QA                 68    0.026   4.773   1.182   0.003   0.718   0.279
B P  1S1QA                 69    0.062   8.741   0.060   0.018   0.846   0.136
B I  1S1QA                 70    0.016   2.960   1.548   0.011   0.918   0.071
B C  1S1QA                 71    0.049   6.894   0.368   0.006   0.962   0.032
B L  1S1QA                 72    0.019   3.461   1.254   0.001   0.900   0.099
B W  1S1QA                 73    0.062  14.839   0.389   0.021   0.756   0.223
B L  1S1QA                 74    0.032   5.951   0.765   0.021   0.451   0.528
B L  1S1QA                 75    0.129  23.565   0.620   0.018   0.088   0.893
E D  1S1QA                 76    0.397  57.193  -0.403   0.058   0.017   0.925
E T  1S1QA                 77    0.442  61.347  -0.288   0.058   0.017   0.925
B Y  1S1QA                 78    0.132  28.166   0.752   0.016   0.005   0.979
E P  1S1QA                 79    0.230  32.651  -0.399   0.016   0.005   0.979
E Y  1S1QA                 80    0.358  76.483  -0.115   0.018   0.019   0.964
E N  1S1QA                 81    0.427  62.498  -0.679   0.005   0.045   0.951
B P  1S1QA                 82    0.144  20.363   0.792   0.004   0.085   0.910
B P  1S1QA                 83    0.044   6.201  -0.911   0.005   0.336   0.660
B I  1S1QA                 84    0.074  13.690   0.742   0.001   0.900   0.099
B C  1S1QA                 85    0.020   2.836   0.978   0.001   0.959   0.040
B F  1S1QA                 86    0.070  13.989   0.234   0.001   0.959   0.040
B V  1S1QA                 87    0.022   3.412   0.275   0.002   0.816   0.182
E K  1S1QA                 88    0.268  55.169   1.001   0.004   0.420   0.576
B P  1S1QA                 89    0.100  14.261  -0.345   0.004   0.085   0.910
E T  1S1QA                 90    0.429  59.447  -0.410   0.018   0.019   0.964
E S  1S1QA                 91    0.574  67.238  -0.637   0.018   0.047   0.935
E S  1S1QA                 92    0.567  66.406  -0.811   0.018   0.047   0.935
B M  1S1QA                 93    0.188  37.679  -0.386   0.022   0.359   0.619
E T  1S1QA                 94    0.414  57.366  -0.766   0.023   0.655   0.322
B I  1S1QA                 95    0.064  11.895  -0.571   0.023   0.655   0.322
E K  1S1QA                 96    0.326  67.140   0.202   0.021   0.451   0.528
B T  1S1QA                 97    0.300  41.610  -1.194   0.004   0.197   0.799
B G  1S1QA                 98    0.294  23.130  -0.738   0.018   0.088   0.893
E K  1S1QA                 99    0.463  95.157  -0.172   0.018   0.088   0.893
B H  1S1QA                100    0.291  52.987  -0.816   0.022   0.359   0.619
B V  1S1QA                101    0.046   7.039  -0.512   0.022   0.552   0.426
B D  1S1QA                102    0.197  28.373   0.141   0.020   0.205   0.775
E A  1S1QA                103    0.502  55.375  -1.635   0.018   0.047   0.935
E N  1S1QA                104    0.575  84.165  -0.543   0.018   0.047   0.935
B G  1S1QA                105    0.088   6.910  -1.666   0.004   0.197   0.799
E K  1S1QA                106    0.218  44.843   0.974   0.003   0.718   0.279
B I  1S1QA                107    0.033   6.179  -0.029   0.001   0.900   0.099
B Y  1S1QA                108    0.177  37.846   0.574   0.018   0.846   0.136
B L  1S1QA                109    0.075  13.696   0.855   0.021   0.451   0.528
B P  1S1QA                110    0.228  32.325   0.160   0.064   0.216   0.721
B Y  1S1QA                111    0.118  25.217  -0.618   0.125   0.227   0.648
B L  1S1QA                112    0.047   8.679  -0.323   0.125   0.227   0.648
E H  1S1QA                113    0.331  60.209  -0.567   0.125   0.227   0.648
E E  1S1QA                114    0.346  60.516  -0.136   0.118   0.150   0.732
B W  1S1QA                115    0.089  21.380   0.211   0.052   0.084   0.864
E K  1S1QA                116    0.493 101.328   0.470   0.018   0.047   0.935
E H  1S1QA                117    0.365  66.412  -0.261   0.018   0.019   0.964
E P  1S1QA                118    0.700  99.316  -0.198   0.018   0.019   0.964
E Q  1S1QA                119    0.518  92.497   0.058   0.018   0.019   0.964
B S  1S1QA                120    0.102  12.001  -0.361   0.058   0.017   0.925
E D  1S1QA                121    0.306  44.095   0.619   0.257   0.016   0.727
B L  1S1QA                122    0.038   7.013   0.001   0.938   0.007   0.055
B L  1S1QA                123    0.129  23.657   0.315   0.970   0.001   0.030
E G  1S1QA                124    0.317  24.956   0.488   0.970   0.001   0.030
B L  1S1QA                125    0.044   8.001   0.218   0.970   0.001   0.030
B I  1S1QA                126    0.027   4.921   0.630   0.970   0.001   0.030
E Q  1S1QA                127    0.324  57.866   1.218   0.970   0.001   0.030
B V  1S1QA                128    0.226  34.690   1.222   0.970   0.001   0.030
B M  1S1QA                129    0.022   4.342   1.356   0.970   0.001   0.030
B I  1S1QA                130    0.114  21.016   1.090   0.988   0.000   0.012
E V  1S1QA                131    0.393  60.373   1.096   0.970   0.001   0.030
B V  1S1QA                132    0.113  17.414   0.974   0.970   0.001   0.030
B F  1S1QA                133    0.032   6.362   0.127   0.923   0.002   0.076
B G  1S1QA                134    0.214  16.873  -0.237   0.694   0.003   0.303
E D  1S1QA                135    0.644  92.858   0.422   0.502   0.002   0.495
E E  1S1QA                136    0.415  72.535   0.175   0.176   0.004   0.820
B P  1S1QA                137    0.220  31.161   0.657   0.016   0.005   0.979
E P  1S1QA                138    0.332  47.139  -1.288   0.005   0.015   0.979
B V  1S1QA                139    0.200  30.755  -1.318   0.018   0.047   0.935
E F  1S1QA                140    0.320  64.224  -0.396   0.018   0.047   0.935
E S  1S1QA                141    0.762  89.271  -1.420   0.003   0.003   0.994

Explain the output.  Go back.
```

---
